# Supplementary material for: A type II protein arginine methyltransferase regulates merozoite invasion in Plasmodium falciparum
Source: Commun Biol. 2023 Jun 22;6:659. doi: 10.1038/s42003-023-05038-z (PMC10287762; doi:10.1038/s42003-023-05038-z)

Supplementary Information

A type II protein arginine methyltransferase regulates merozoite invasion in *Plasmodium falciparum*

Amuza Byaruhanga Lucky[1#], Chengqi Wang[2#], Min Liu[1,3#], Xiaoying Liang[1], Hui Min[1], Qi Fan[4], Faiza Amber Siddiqui[1], Swamy Rakesh Adapa[2], Xiaolian Li[1], Rays Jiang[2], Xiaoguang Chen[3], Liwang Cui[1], Jun Miao[1*]

[1] Department of Internal Medicine, Morsani College of Medicine, University of South Florida, Tampa, FL 33612, USA

[2] Center for Global Health and Infectious Diseases, Department of Global Health, University of South Florida, Tampa, FL 33612, USA

[3] Department of Pathogen Biology, School of Public Health, Southern Medical University, Guangzhou, Guangdong 510515, China

[4] Dalian Institute of Biotechnology, Dalian, Liaoning, China

[#] These authors contributed equally to this work

[*] Corresponding author

Jun Miao

jmiao1@usf.edu

Supplementary Figure 1-11

a

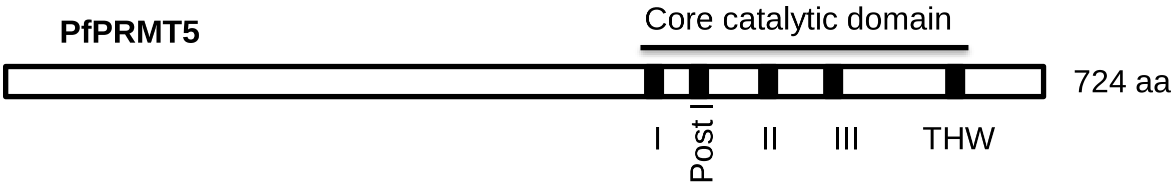

b

```
               #
TbPRMT5 ASGVYEVFEQDRTKYQQYHTAMSKYFNEWLNHSESRSHEKMWLQGPNPRDG---CGCSAM 428
PfPRMT5 SSQTYEVFEKDRKKYEQYELATSKYLSNWKKGKKINNKNKQSNKNKNNNNNNNMEEQQND 442
HsPRMT5 ESQTYEVFEKDPIKYSQYQQAIYKCLLDRVP-----------------------EEEKDT 356
SpSKB1  ENITYEIFERDPVKYAQYEQAIFSALMDRDE----------------------------S 343
              . .*: **:*  **  *  *    : :

          I                   Post I
TbPRMT5 YVVLLGAGRGPLISECLCAATGV-GVRVHLFVVEKNPEALELVRLRVRADPQWHDWMNYS 490
PfPRMT5 TIFVVGAGRGPLVDTTLSALQKNEMTDYEIYAIEKNDSAIIILNNRVQTE--EWK----- 498
HsPRMT5 VLMVLGAGRGPLVNASLRAAKQA-DRRIKLYAVEKNPNAVVTLENWQFEE--WGS----- 411
SpSKB1  RIAVVGAGRGPLVDCALRAAISS-SRTVDMIALEKNPNAFSMLLMRNRQD--WAG----- 398
         : ::*******:.  * *        .: .:*** .*.  :      :

                                          II  Δ        Δ
TbPRMT5 GHVVETIYADGRSVWSGEAPGSDDRLPPYWGLCDLVVSELLGSFGDNELSPECLDDFYCN 550
PfPRMT5 --NVKVIHSDIRY-L------------DIPKKADIIISELLGSFGDNELFPECMDGIKKF 543
HsPRMT5 --QVTVVSSDMRE-W------------VAPEKADIIVSELLGSFADNELSPECLDGAQHF 456
SpSKB1  --KVTLVFGDMRT-W------------NPDYKIDILVSELLGSMGDNELSPECLDGVQHV 443
           *  : .* *                *:::******:.**** ***:*.
             III
TbPRMT5 LLSYQESSGIPCNPYLTSIPQQYTAWIAPLHSARMEESVATAAFGGLTTPPADCHDRHAA 610
PfPRMT5 LKD----DG-------ISIPMNCVSYLEPISCSALYHKVMENNI--------------SG 578
HsPRMT5 LKD----DG-------VSIPGEYTSFLAPISSSKLYNEVRACREKDRD-P--------EA 496
SpSKB1  LDE---ETG-------ICIPSSYISYVTPIMSPKLWSEARNMND--------------PN 479
        *  .     *        .** .  ::: *:  .  :  ..

TbPRMT5 LYHSMFVSNVCR-----AVGLCLPQPCWTFHHFATKV------QSKEREATLNFTLSGDG 659
PfPRMT5 GNESFYVVNMYSYTKISQES---SKECFFFQVPPIHT--KQDNSHNYRYKNINFKIKMDT 633
HsPRMT5 QFEMPYVVRLHNFHQLSAP-----QPCFTFSHPNRDPM-----IDNNRYCTLEFPVEVNT 546
SpSKB1  AFERQYVVLMNSFDFLAADDEFRFQSLWSFHHPNKDSEVYTKNLHNKRFASVRFQASSPG 539
          .  :*  :              :  : *     .        : *  :.:.*  .

                                              THW loop
TbPRMT5 RFSGFICYFSAVLFTPGDTGNVEDSIALLCASAGSLSTVQYGRTTGLFSWFPAFLPVEPR 719
PfPRMT5 YIHGFLCYFKSQLYDDVYISIE--------------PK---THTPNLHSWFPLYIPINKI 676
HsPRMT5 VLHGFAGYFETVLYQDITLSIR--------------PE---THSPGMFSWFPILFPIKQP 589
SpSKB1  ILHGFAGYFEATLYKDISLSIM--------------PATMEAKSPDMFSWFPIYMPIKKP 585
          : **  **.: *:     .                 :: .:.****  :*::
```

Supplementary Figure 1. PfPRMT5 is a type II PRMT with conserved methyltransferase domains. a Schematic representation of PfPRMT5 showing the conserved core catalytic domains (motifs I, Post I, II, III and THW) and a long non-conserved N-terminus. **b** Amino acid sequence alignment of methyltransferase domains among four PRMT5 from *P. falciparum* (PfPRMT5),*Trypanosoma brucei* (TbPRMT5; GenBank Q38CH6), *Homo sapiens* (HsPRMT5; GenBank O14744), *and Saccharomyces pombe* (SpSKB1; GenBank P78963). Asterisks (*) indicate fully conserved residues, colons (:) indicate conservation between groups of strongly similar properties, and periods (.) indicate conservation between groups of weakly similar properties. The AdoMet binding domain (motifs I, Post I, II, and III) and the THW domain are indicated above the sequences. The double-E loop is marked as triangles. The conserved phenylalanine (F) critical for Type II enzyme is marked with a #.

a

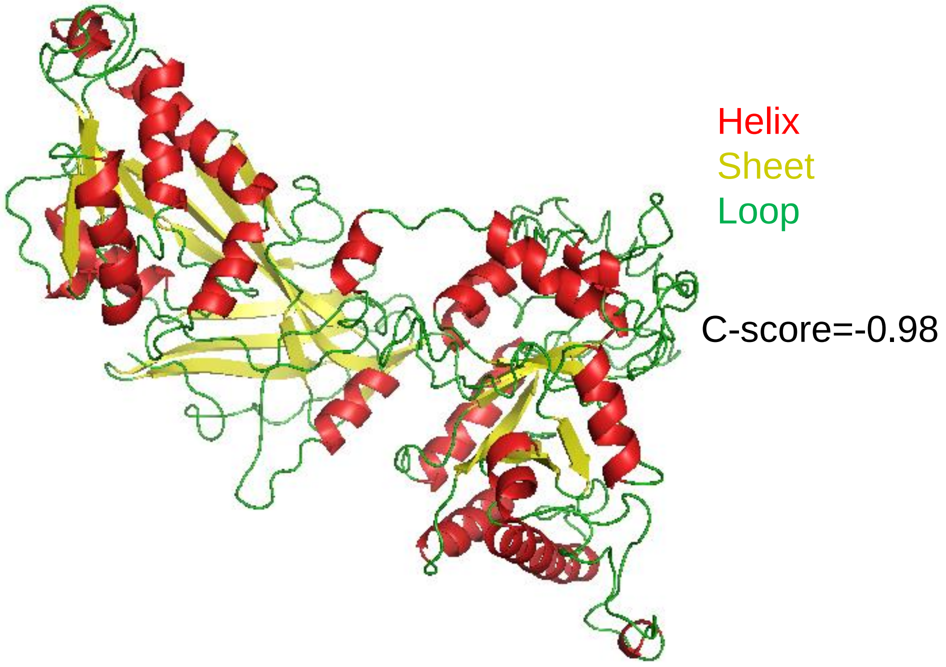

b

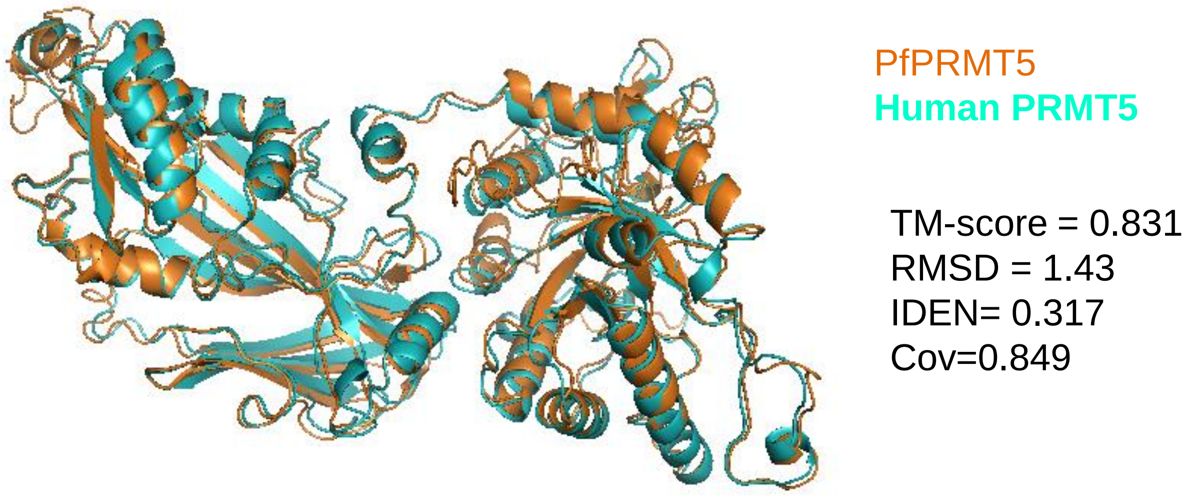

Supplementary Figure 2. PfPRMT5 structure predicted using iTASSER. a PfPRMT5 structure predicted using iTASSER. C-score is typically in the range of [-5,2], where a C-score of higher value signifies a model with a high confidence and vice-versa. **b** Alignment of PfRMT5 with crystal structure of human PRMT5 (4gqbA). TM-score is a metric for measuring the structural similarity of two protein models. TM-score has the value in (0,1], where 1 indicates a perfect match between two structures. Following strict statistics of structures in the PDB, scores below 0.17 corresponds to randomly chosen unrelated proteins whereas with a score higher than 0.5 assume generally the same fold in SCOP/CATH. TM-score is designed to solve two major problems in the traditional metrics such as root-mean-square deviation (RMSD): (1) TM-score measures the global fold similarity and is less sensitive to the local structural variations; (2) magnitude of TM-score for random structure pairs is length-independent. IDEN is the percentage sequence identity in the structurally aligned region. Cov represents the coverage of the alignment by TM-align and is equal to the number of structurally aligned residues divided by length of the query protein.

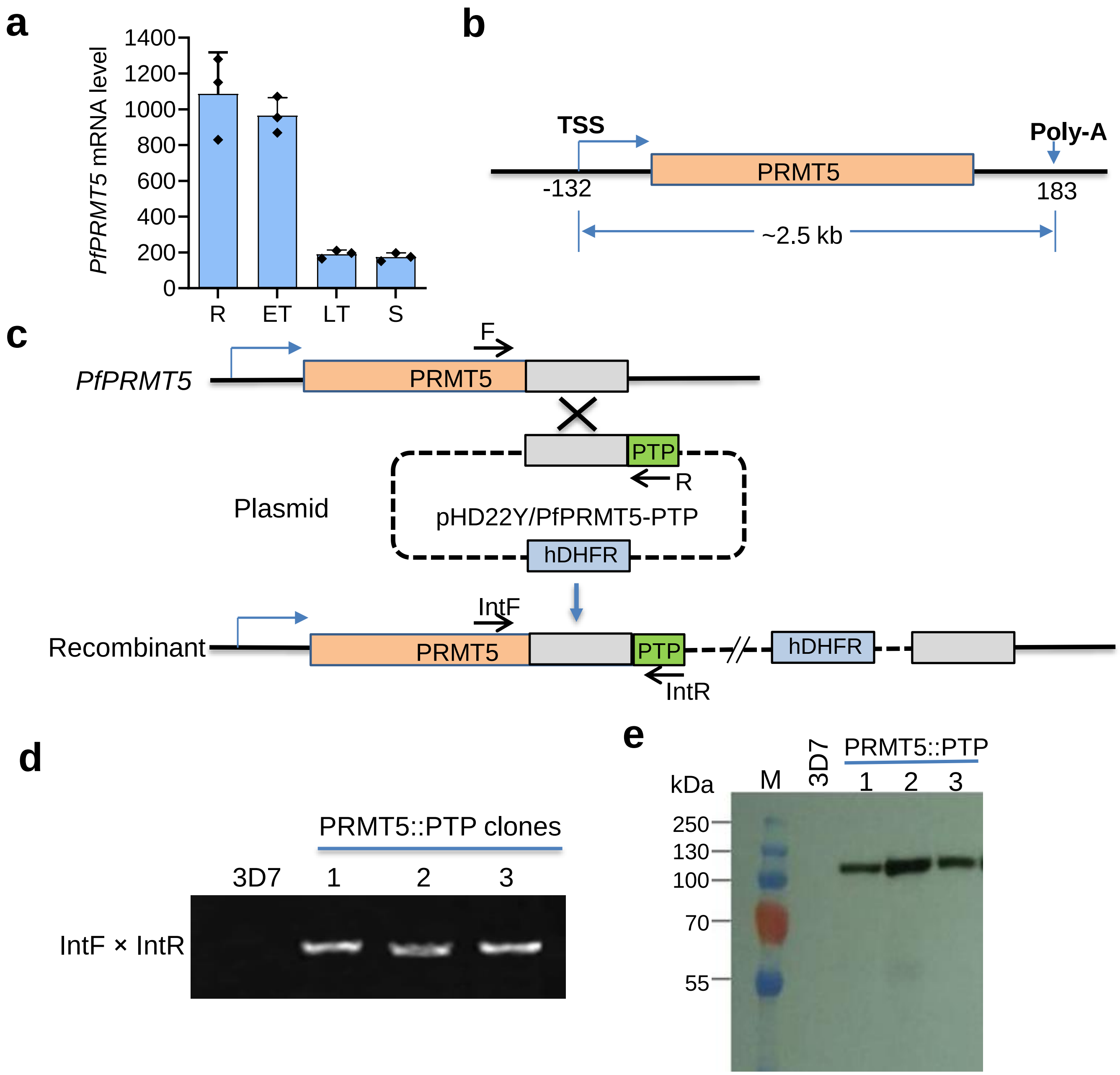

Supplementary Figure 3. *PfPRMT5* transcription and its C-terminal PTP tagging. a *PfPRMT5* mRNA level at different stages of the IDC determined by qRT-PCR. The *seryl-tRNA synthetase* gene was used as the internal reference. R, ring; ET, early trophozoite; LT, late trophozoite; S, schizont. The standard deviation was shown as error bars. **b** Schematic of *PfPRMT5* gene showing the transcription start site (TSS) and polyadenylation site (Poly-A) determined using RACE. The *PfPRMT5* transcript size is estimated to be ~2.5 kb. **c** Schematic plot shows the predicted integration event of PTP tag fusion at the endogenous *PfPRMT5* locus. Top: *PfPRMT5* locus. The box represents the exon. Grey boxes indicate the region used for homologous recombination. Middle: the plasmid pHD22Y/PRMT5-PTP. Bottom: The resultant single-crossover event at the *PfPRMT5* locus. The arrows indicate the primers (IntF and IntR) for integration-specific PCR in D. **d** PCR identification of PTP-tagged clones (1-3). **e** Western blot with anti-protein C antibody detecting the PTP-tag. The bands at ~106 kDa were consistent with the size of the PfPRMT5-PTP fusion protein (Lanes 1-3). M, protein molecular marker in kDa.

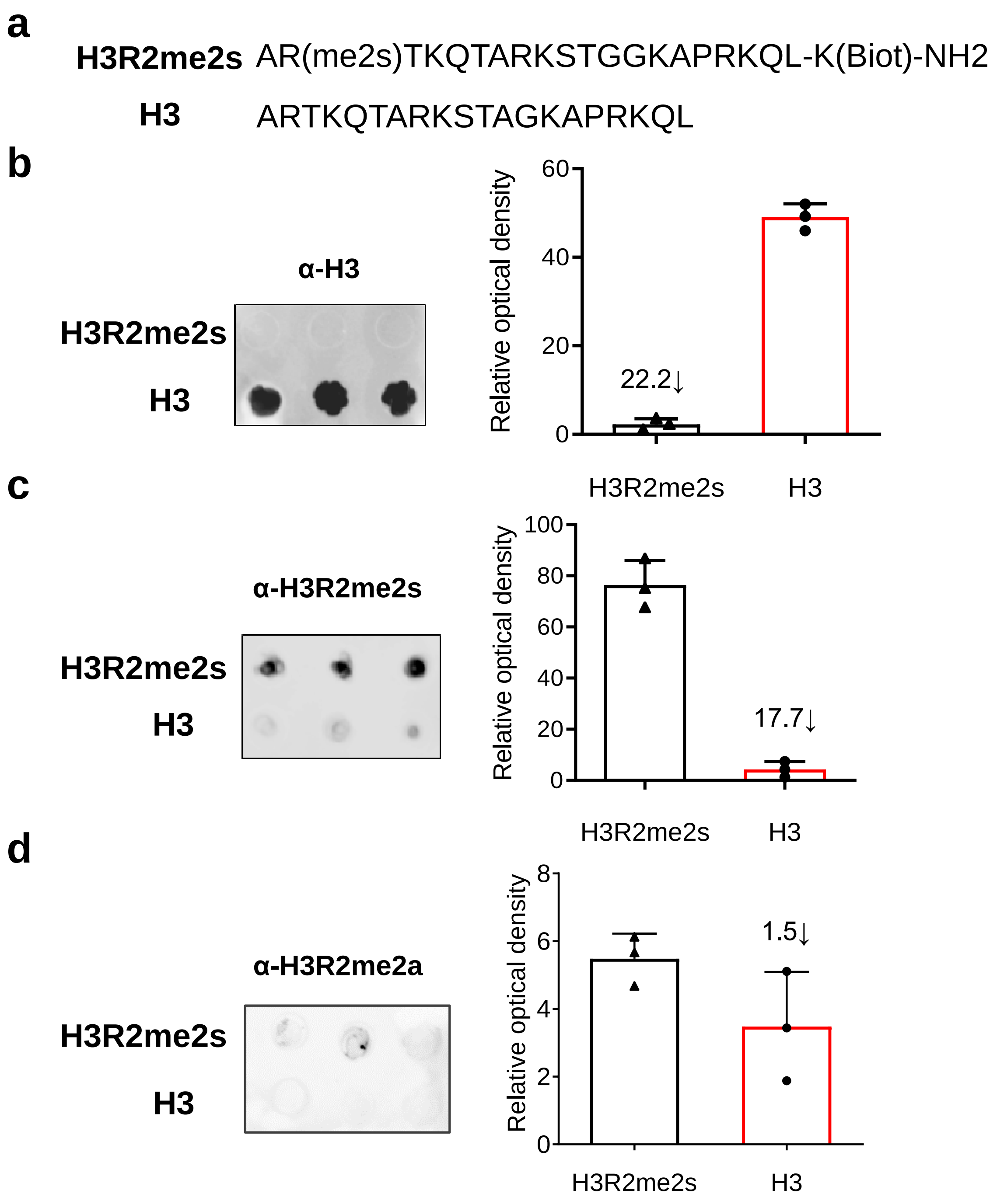

Supplementary Figure 4. Verification of the H3R2me2s antibodies. a H3 and modified H3 peptides used in dot blot analysis. **b** Dot blot analysis of anti-H3 antibodies to specifically detect unmodified histone H3 peptides but not modified H3 peptides (R2me2s). Relative optical density was calculated to quantify the degree to which the antibodies detect each peptide. The α-H3 antibodies had a ~22-fold greater specificity towards the corresponding unmodified peptide than the R2me2s modified peptide. **c** Dot blot analysis of anti-H3R2me2s antibodies to specifically detect modified histone H3 peptides (R2me2s) but not unmodified H3 peptides. The α-H3R2me2s antibodies had an ~18-fold greater specificity towards the Rme2s modified peptide than the unmodified peptide. **d** Dot blot analysis to show the degree to which anti-H3R2me2a antibodies cross-react with modified histone H3 peptides (R2me2s) and unmodified H3 peptides. anti-H3R2me2a antibodies did not show substantial cross-reaction to either peptides as in **a** & **b**. anti-H3R2me2a was ~1.5-fold more unspecific to H3Rme2s peptides than unmodified H3 peptides. Each dot was spotted with 250 ng of the respective peptides. All antibodies were used at 1:5000 dilution. The standard deviation was shown as error bars.

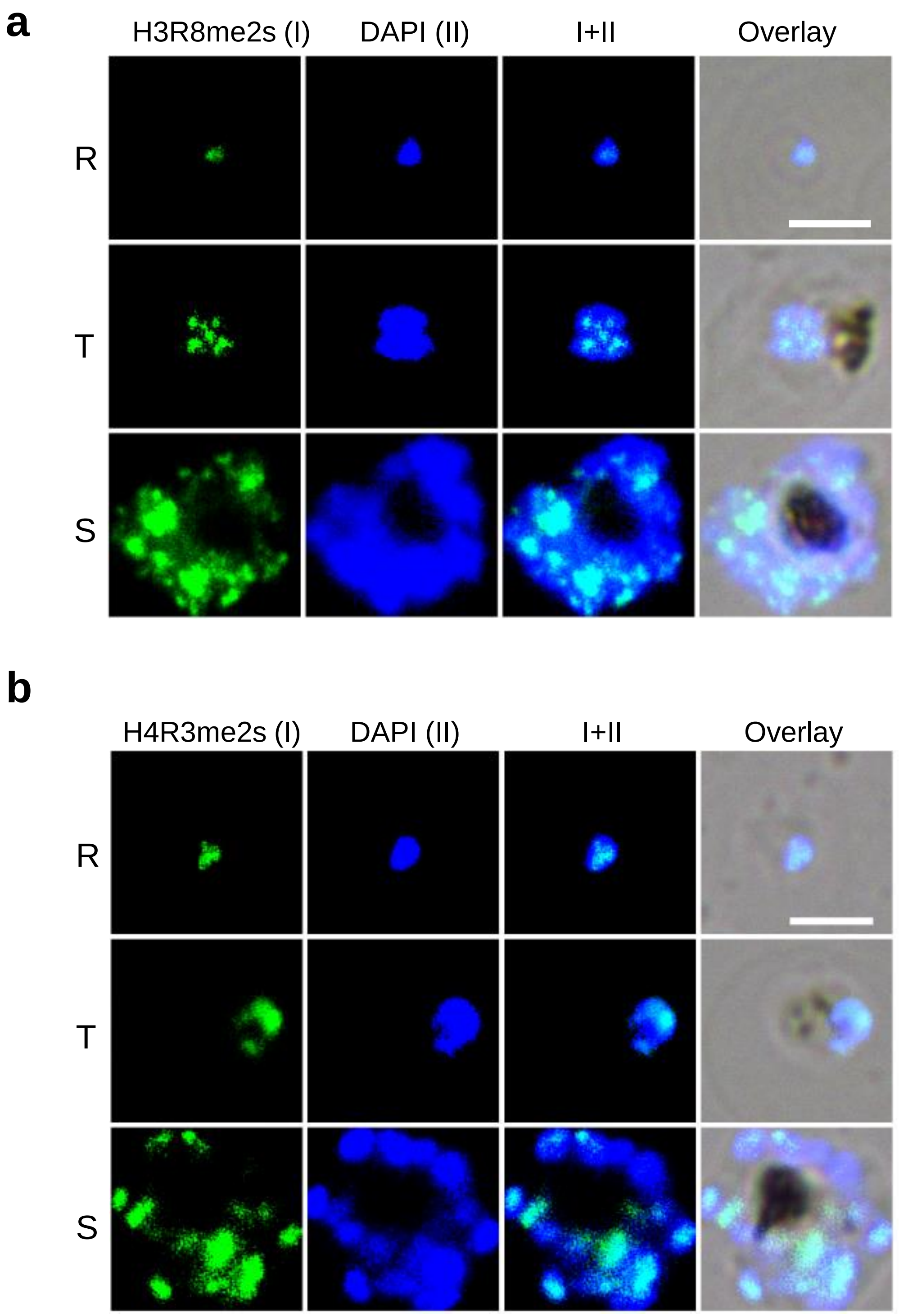

Supplementary Figure 5. Localization of H3R8me2s and H4R3me2s in the parasites. **a** and **b** IFA with anti-H3R8me2s (**a**) and H4R3me2s (**b**) showing the localization of these marks during asexual development. Alexa fluor 488-conjugated anti-rabbit IgG as the secondary antibodies. Nuclei were stained with DAPI. The size of the scale bar is 5 μM. R: ring, T: trophozoite, and S: schizont.

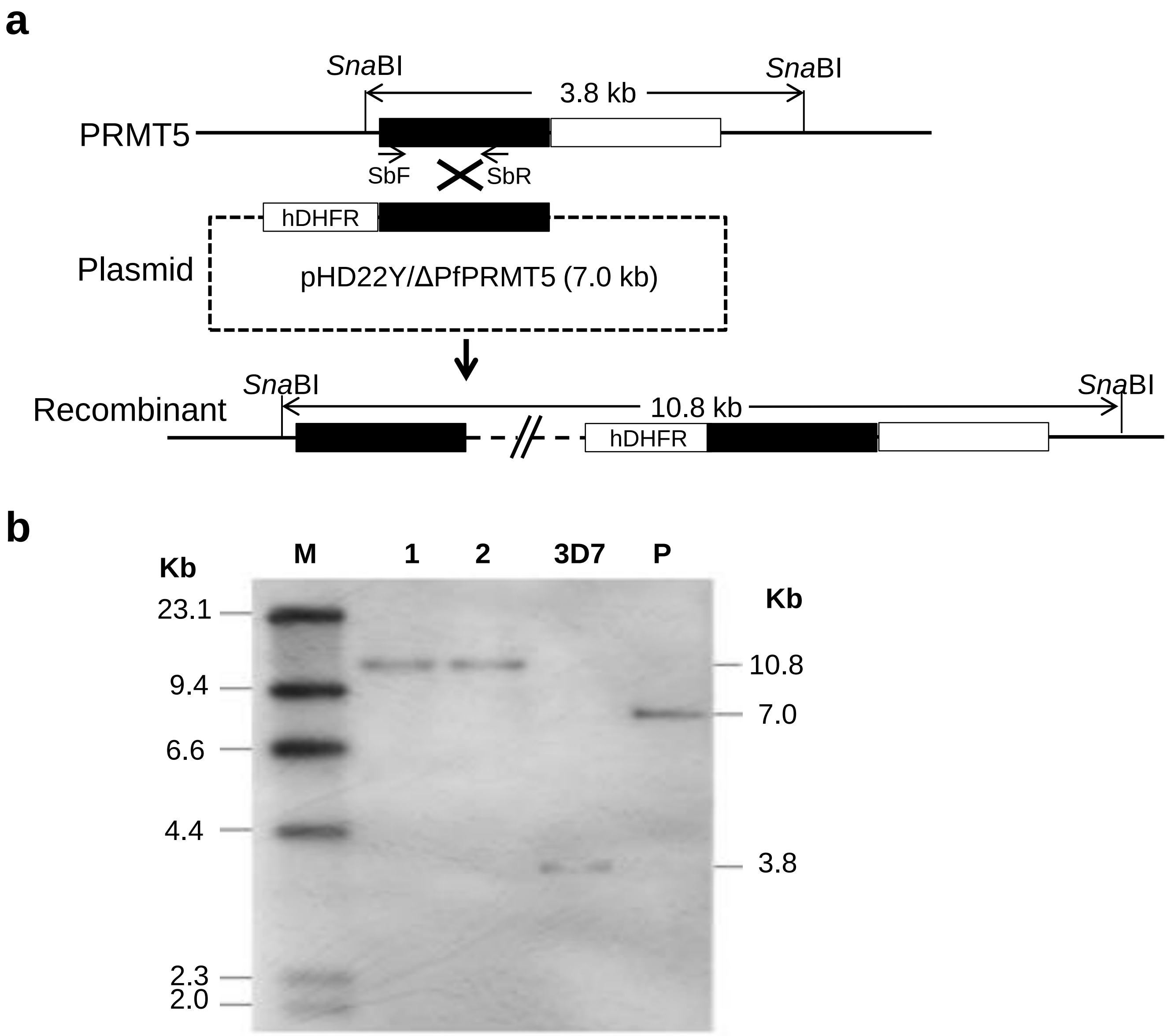

Supplementary Figure 6. Disruption of *PfPRMT5* by single crossover recombination. **a** Schematic diagram represents the predicted disruption of *PfPRMT5* by a single-crossover event. Top: *PfPRMT5* locus on chromosome 13. Middle: the transfection plasmid pHD22Y/ΔPfPRMT5 showing the *PfPRMT5* genomic fragment for homologous recombination and the drug selection cassette (human DHFR). Bottom: the predicted single-crossover event at the *PfPRMT5* locus showing the integration of one copy of the plasmid. Restriction enzyme *Sna*BI sites and the expected sizes of DNA fragments after *Sna*BI digestion are shown. **b** Confirmation of *PfPRMT5* gene disruption by Southern blot. Genomic DNA from wildtype 3D7, one clone each from two transfections (Lanes 1 and 2) and the plasmid (P) were digested with *Sna*BI and separated in a 0.8% agarose gel. The blot was hybridized to DIG-labeled PCR product using primers SbF x SbR shown in **(a)**. The ~10.8 kb bands in Lanes 1 and 2 indicate that these two clones had predicted disruption of *PfPRMT5*. M, DNA markers in Kb.

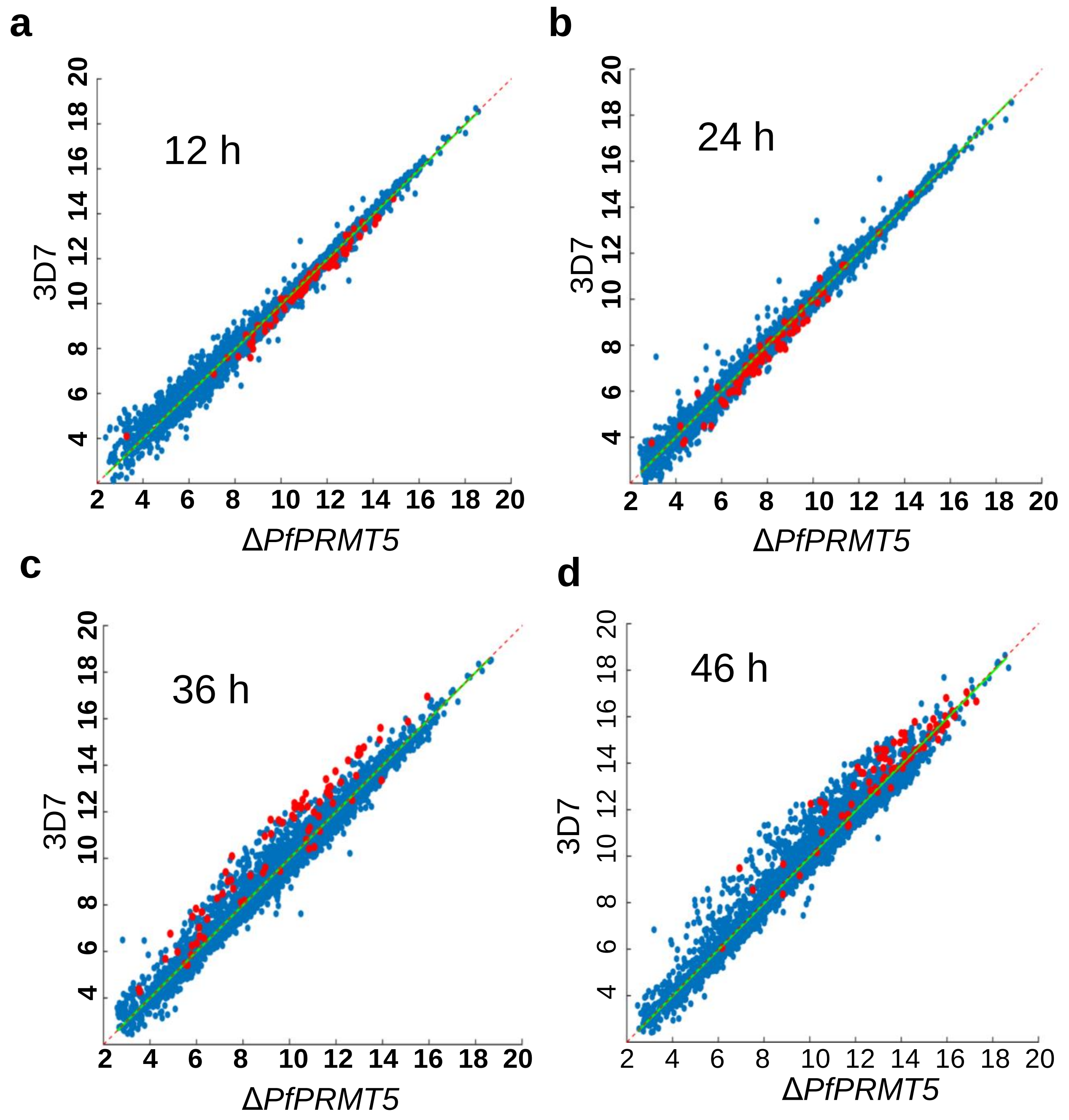

Supplementary Figure 7. Comparison of transcriptomes between *ΔPfPRMT5* and wildtype 3D7 during IDC. a-d The transcriptional levels of each gene were shown as the $\log_2$ values from RNA-seq analysis at 12 (**a**), 24 (**b**), 36 (**c**), and 46 (**d**) hpi. 76 invasion-related genes are shown as red dots.

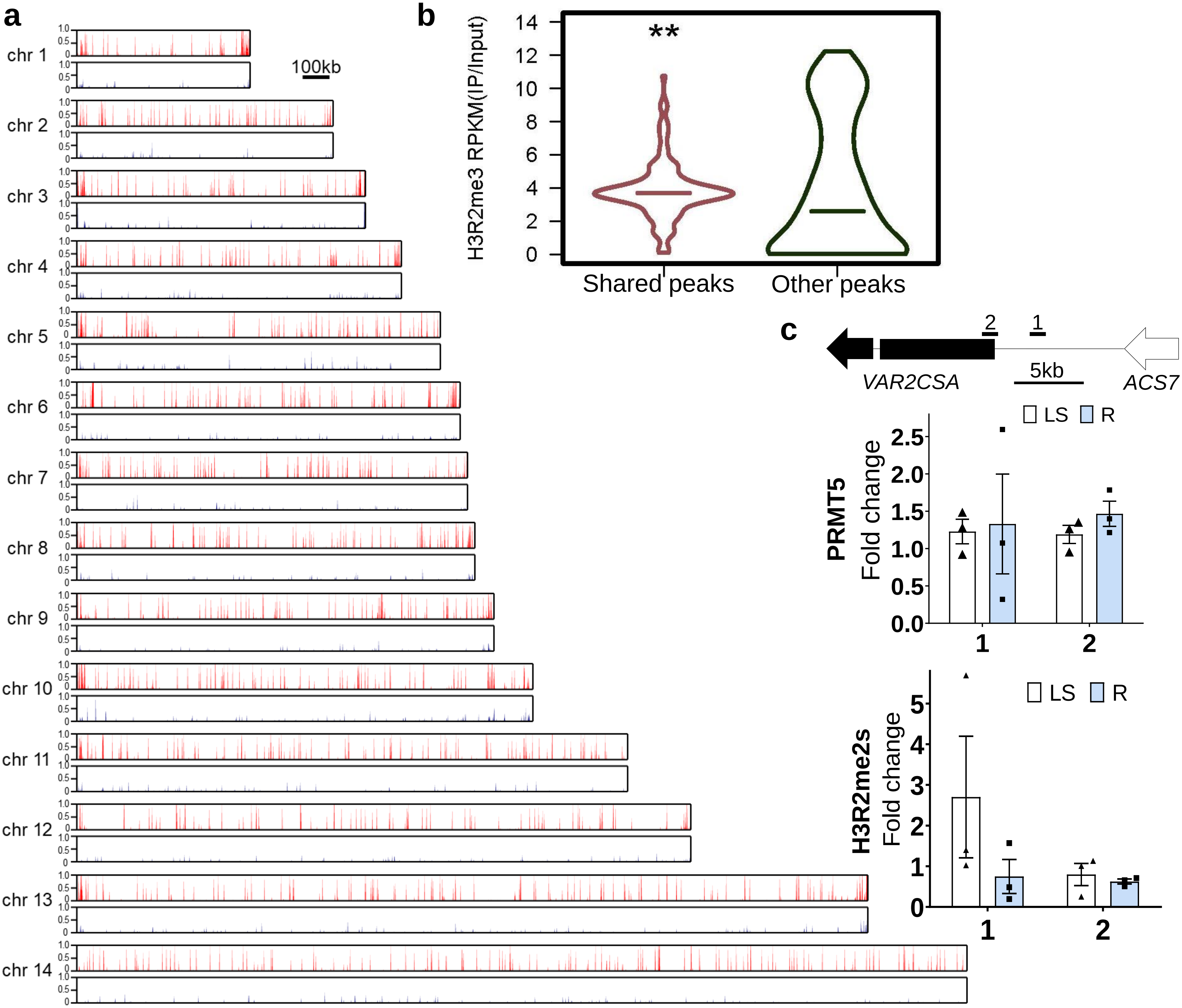

Supplementary Figure 8. H3R2me2s chromatin landscape, shared peaks between H3R2me2s and PfPRMT5, and ChIP-qPCR of PfPRMT5 and H3R2me2s in *VAR2CSA*. a Normalized raw data showed that significantly higher signals were identified from H3R2me2s (in red) than IgG control (in blue) in 14 chromosomes. The signals were normalized based on RPKM (reads per kilobase of transcript per million reads mapped). **b** Violin plots indicate that the signals of H3R2me2s in shared peaks between PfPRMT5 and H3R2me2s in 5'UTR regions were significantly higher than the rest of H3R2me2s peaks in the other 5'UTRs (Wilcoxon test, p <0.01). **c** The enrichment of PfPRMT5 and H3R2me2s were determined by ChIP-qPCR using primer pairs marked as 1 and 2 located in the promoters of *VAR2CSA* at ring (R) and late schizont (LS). The fold change indicates the enrichment relative to the reference gene *seryl-tRNA synthetase* (PF3D7_0717700). The standard deviation was shown as error bars.

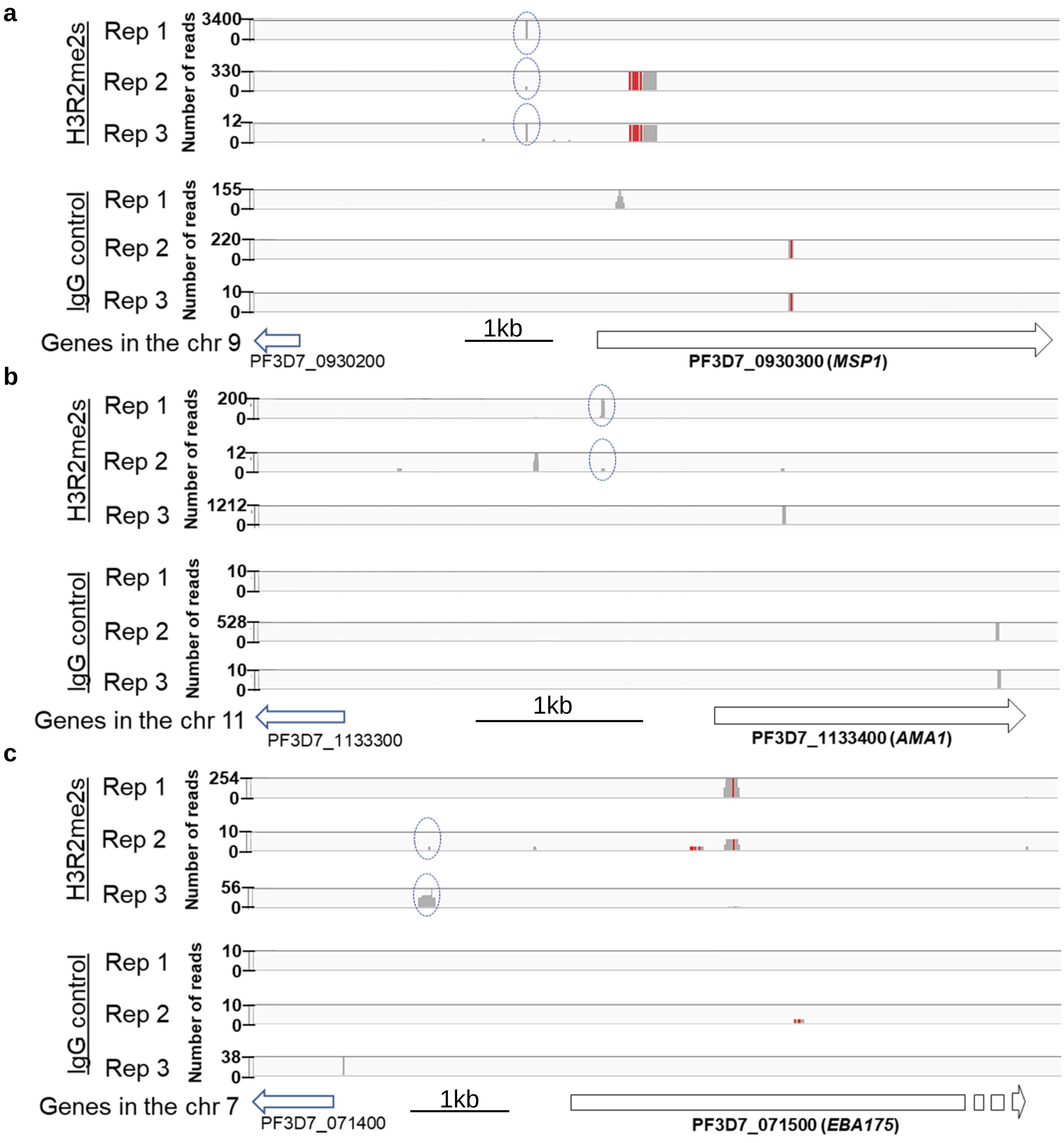

Supplementary Figure 9. H3R2me2s signals in the 5'UTRs of *MSP1*, *AMA1* and *EBA175*. a-c The enrichment of H3R2me2s signals (circled in the figures) in the 5'UTR regions of *EBA175*, *MSP1*, and *AMA1* was detected by H3R2me2s CUT&Tag-seq. The peaks in the 5'UTRs of *MSP1* (**a**), *AMA1* (**b**),and *EBA175* (**c**) in the three replicates (Rep 1-3) of CUT&Tag-seq for H3R2me2s and their IgG control were shown by Integrative Genomics Viewer (IGV).

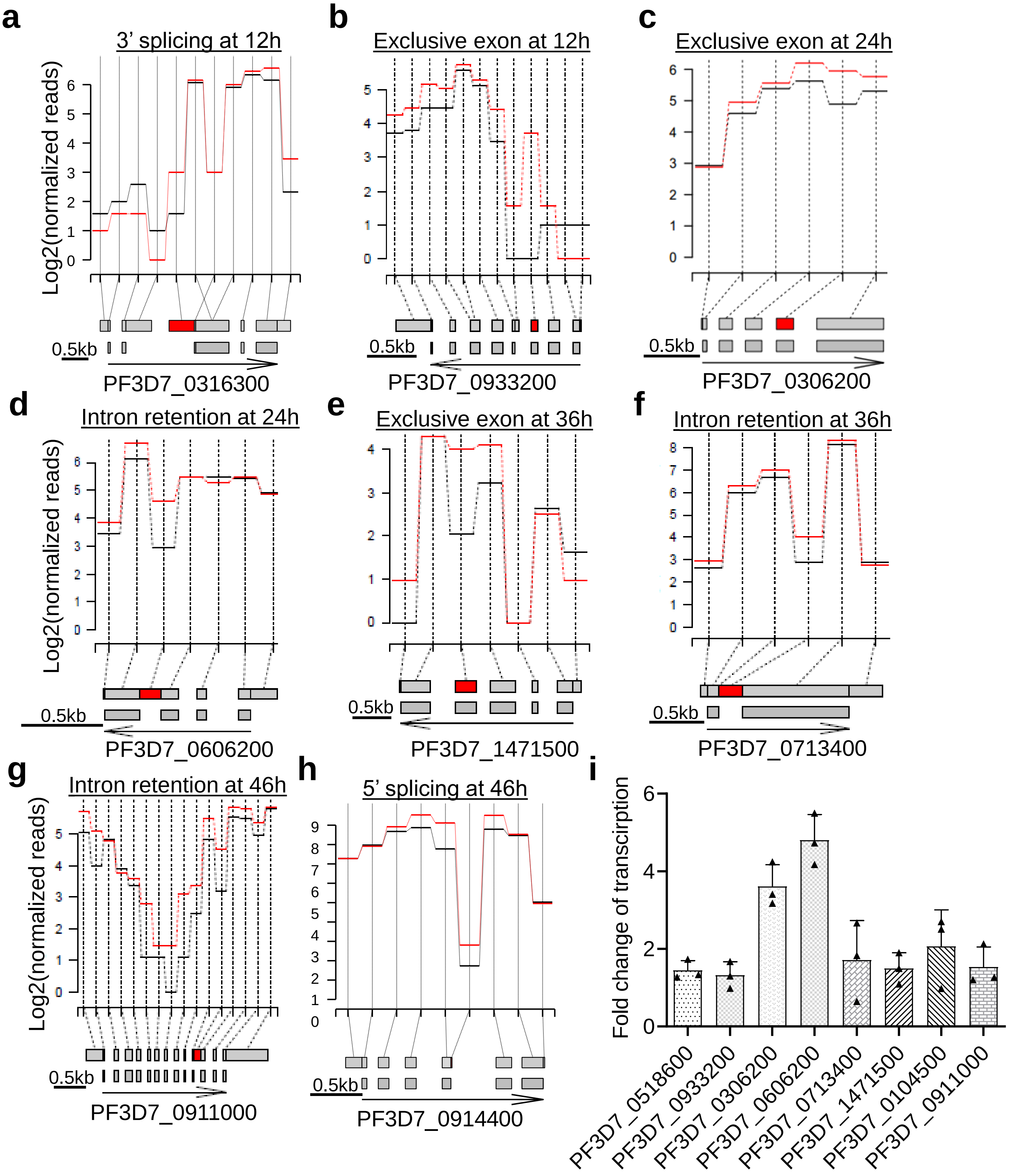

Supplementary Figure 10. Verification of the alternative splicing events in *ΔPfPRMT5*. a-h. Eight identified altered alternative splicing events upon *PfPRMT5* disruption. The annotated exon/intron from known genome model and transcriptional bins identified from RNA-seq were depicted at the bottom of each diagram with red bin denoting altered alternative splicing events. The expression levels of each transcriptional bins in *ΔPfPRMT5* and wildtype parasite were shown as the red and black curves in the diagram, respectively. **i** Transcription of alternative splicing events was analyzed by RT-qPCR and fold changes of alternative splicing transcription in upon *PfPRMT5* disruption compared to wildtype parasites were shown in bar graph. The standard deviation was shown as error bars.

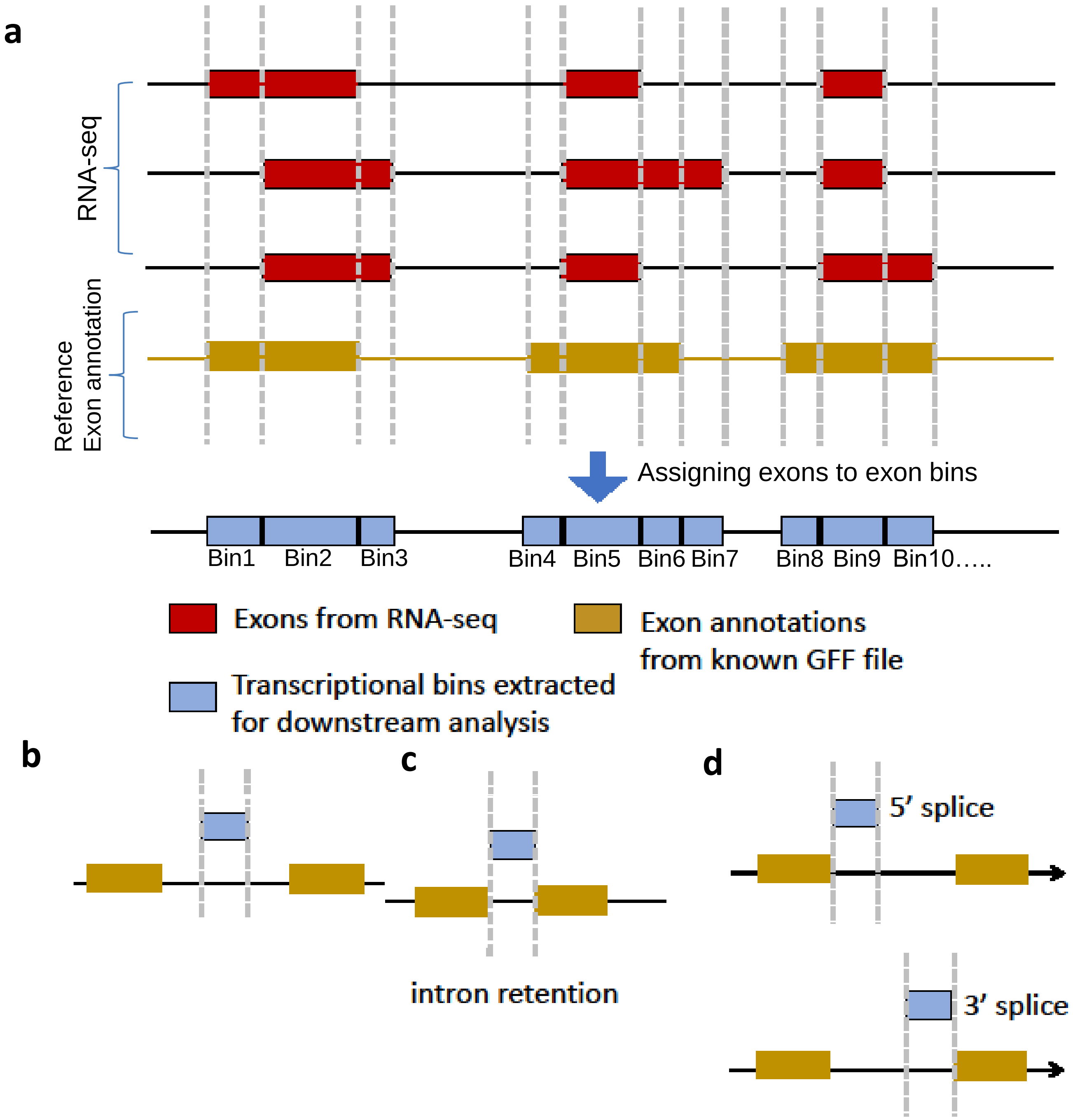

Supplementary Figure 11. Program design to detect alternative RNA splicing events. **a** Transcripts from RNA-seq (red exons) were compared with the reference annotated exons (brown) in *P. falciparum* genome (PlasmoDB version 39.0) using a custom python script, 'geneInfPick.py'. If an exon from the RNA-seq data did not match the respective annotated exon, this exon was then cut into two or multiple segments (transcriptional bins). The differential expression of these alternative splicing transcripts between 3D7 and ΔPfPRMT5 were analyzed by DEXseq and Deseq. **b-d.** A custom python script "alternativeSplicing_annotaiton.py" was used to assign the type of alternative splicing events (**b**: exon skipping; c: intron retention; **d**: 5' or 3' alternative splicing) for each transcriptional bin.

Supplementary Figure 12: Original blot/gel images corresponding to the figures in the paper

Part 1. Original blot/gel images corresponding to the figure 1a, 1b, 1d, 1e, 1f.

Part 2. Original blot images corresponding to the figure 2e and 2f.

Part 1.Original blot/gel images corresponding to the figure 1a, 1b, 1d, 1e, 1f.

Fig. 1a

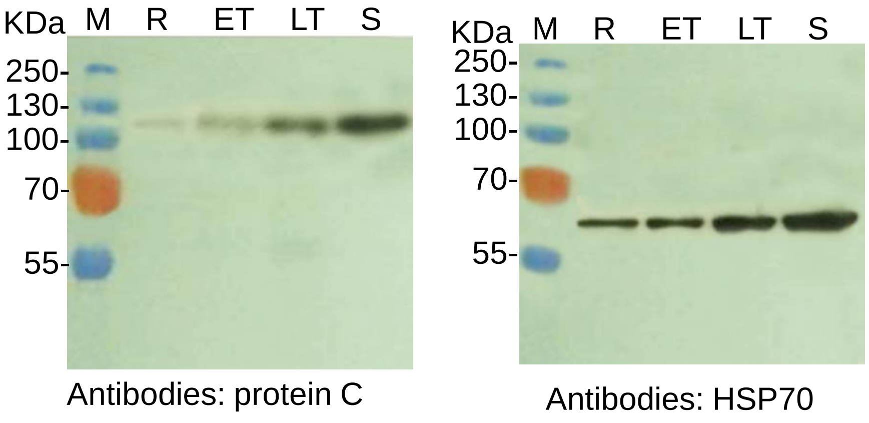

M: protein marker

Fig. 1b

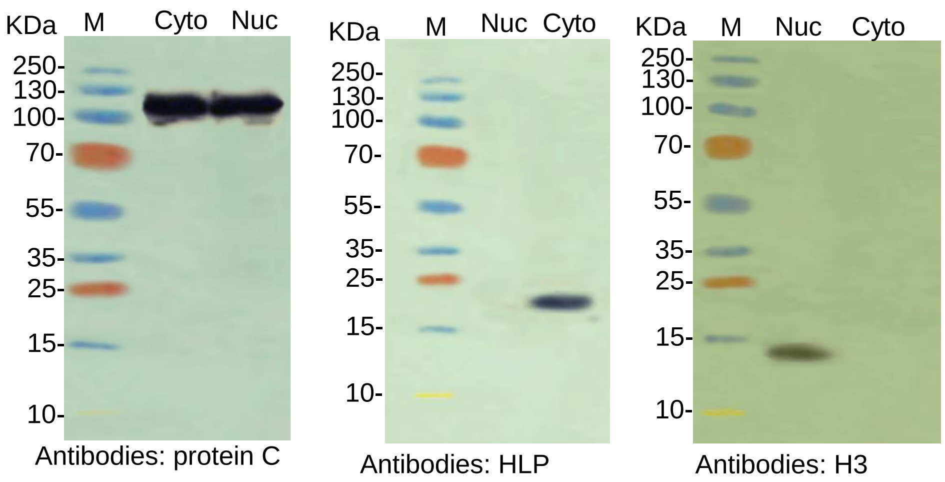

Fig. 1d

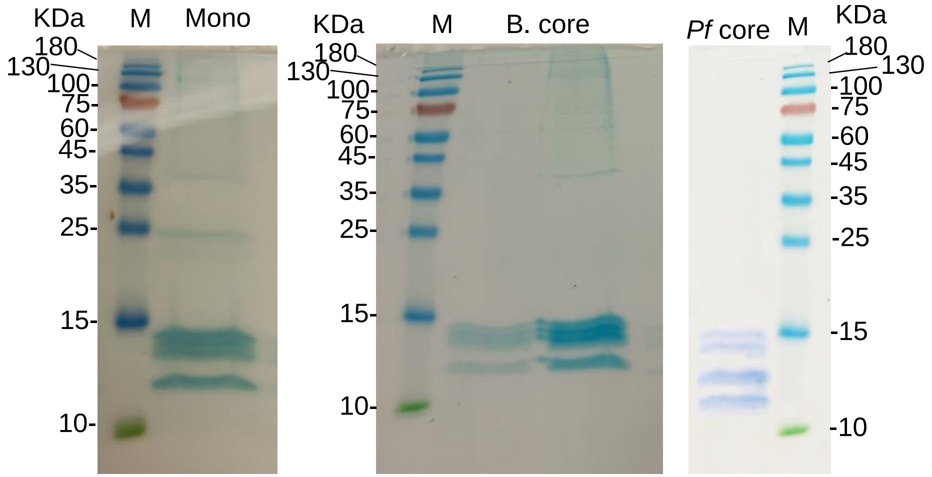

Mono: mononucleosome
B. core: bovine core histones
Pf core: Pf core histones

Fig. 1e

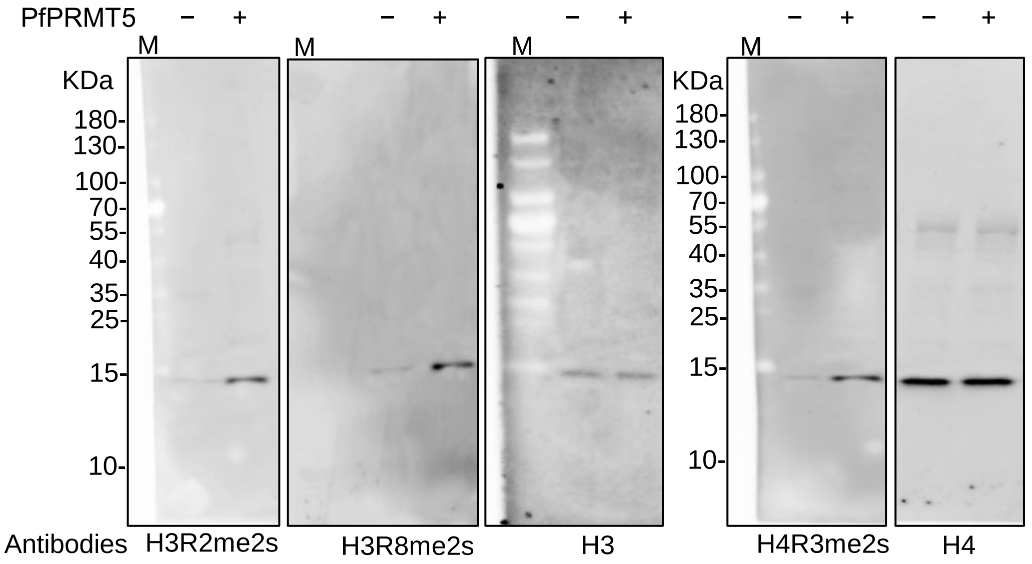

Fig. 1f

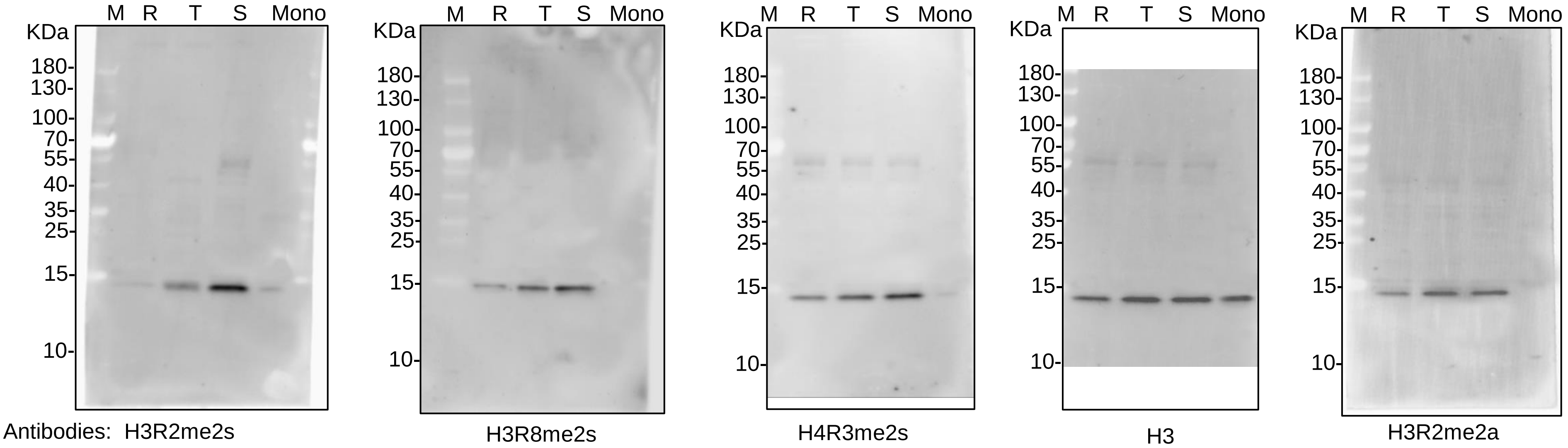

M: protein marker; Mono: mononucleosome assembled from recombinant human histones without any PTM

Part 2. Original blot images corresponding to the figure 2e and 2f.

Fig. 2e

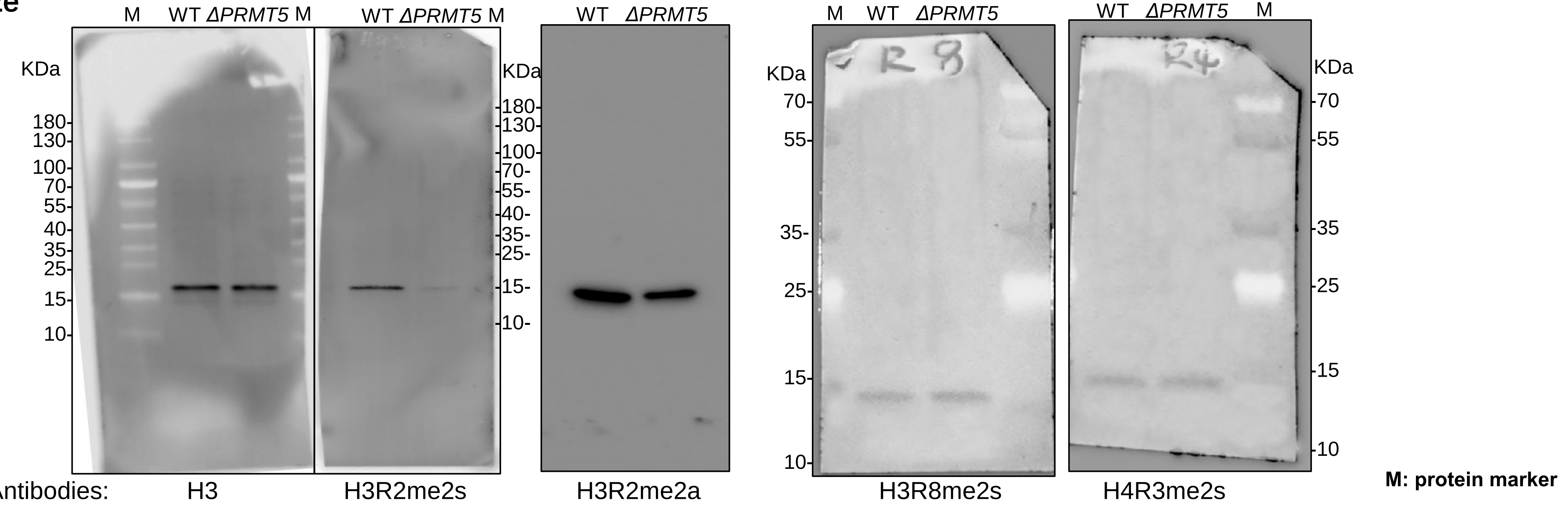

Fig. 2f

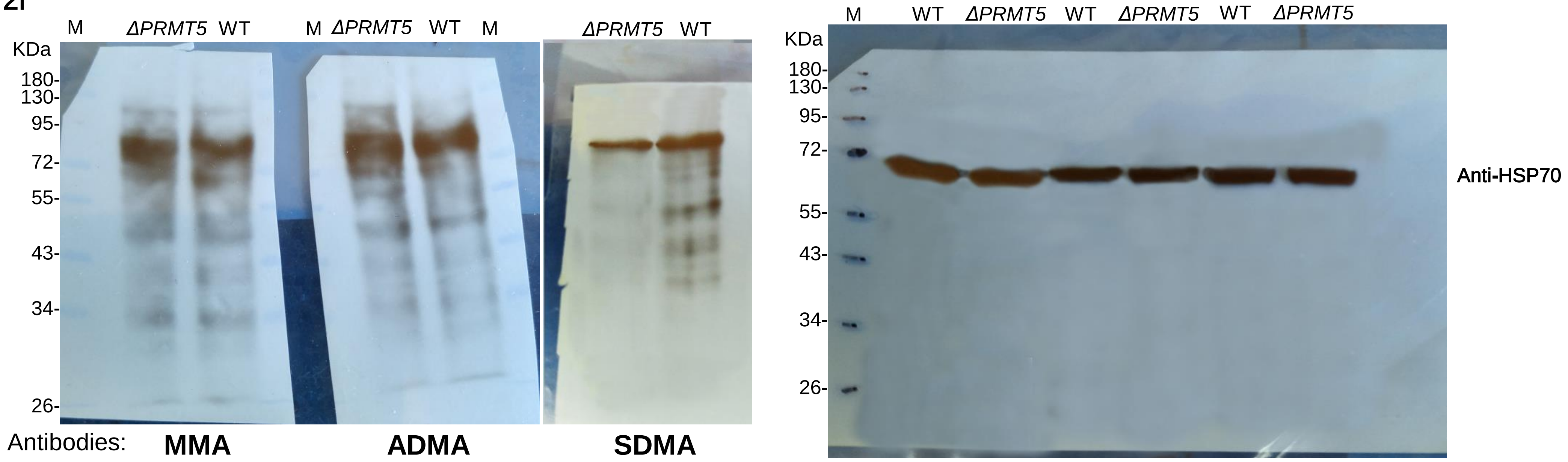

Supplement: Supplementary file 2 — Supplementary Information [file 42003_2023_5038_MOESM2_ESM.pdf]
