# Supplementary material for: A type II protein arginine methyltransferase regulates merozoite invasion in Plasmodium falciparum
Source: Commun Biol. 2023 Jun 22;6:659. doi: 10.1038/s42003-023-05038-z (PMC10287762; doi:10.1038/s42003-023-05038-z)
Supplement: Supplementary file 3 — Description of Additional Supplementary Files [file 42003_2023_5038_MOESM3_ESM.pdf]

Description of Additional Supplementary Files

File name: Supplementary Data 1
Description: Expression microarray data of ΔPfPRMT5 and WT During IDC.

File name: Supplementary Data 2
Description: H3R2me2s chromatin landscape at the schizont stage by CUT&Tag-seq

File name: Supplementary Data 3
Description: PfPRMT5 chromatin landscape at the schizont stage by CUT&Tag-seq

File name: Supplementary Data 4
Description: Identification of PfPRMT5-associated proteins.

File name: Supplementary Data 5
Description: The alternative splicing events in ΔPfPRMT5 and WT During IDC based on RNA-seq.

File name: Supplementary Data 6
Description: Known PRMT5 substrates from the published data.

File name: Supplementary Data 7
Description: List of primers in this study.

File name: Supplementary Data 8
Description: The source data behind Figure 2a-d and 5b-c, Supplementary Figure 3a, 4b-d, 8c, and 10i.
