# Supplementary material for: A type II protein arginine methyltransferase regulates merozoite invasion in Plasmodium falciparum
Source: Commun Biol. 2023 Jun 22;6:659. doi: 10.1038/s42003-023-05038-z (PMC10287762; doi:10.1038/s42003-023-05038-z)
Supplement: Supplementary file 12 — Reporting Summary [file 42003_2023_5038_MOESM12_ESM.pdf]

nature portfolio

Corresponding author(s): Jun Miao

Last updated by author(s): Jun 2, 2023

Reporting Summary

Nature Portfolio wishes to improve the reproducibility of the work that we publish. This form provides structure for consistency and transparency in reporting. For further information on Nature Portfolio policies, see our Editorial Policies and the Editorial Policy Checklist.

Statistics

For all statistical analyses, confirm that the following items are present in the figure legend, table legend, main text, or Methods section.

n/a	Confirmed	
☐	☒	The exact sample size (*n*) for each experimental group/condition, given as a discrete number and unit of measurement
☐	☒	A statement on whether measurements were taken from distinct samples or whether the same sample was measured repeatedly
☐	☒	The statistical test(s) used AND whether they are one- or two-sided<br>*Only common tests should be described solely by name; describe more complex techniques in the Methods section.*
☐	☒	A description of all covariates tested
☐	☒	A description of any assumptions or corrections, such as tests of normality and adjustment for multiple comparisons
☐	☒	A full description of the statistical parameters including central tendency (e.g. means) or other basic estimates (e.g. regression coefficient) AND variation (e.g. standard deviation) or associated estimates of uncertainty (e.g. confidence intervals)
☒	☐	For null hypothesis testing, the test statistic (e.g. *F*, *t*, *r*) with confidence intervals, effect sizes, degrees of freedom and *P* value noted<br>*Give P values as exact values whenever suitable.*
☒	☐	For Bayesian analysis, information on the choice of priors and Markov chain Monte Carlo settings
☒	☐	For hierarchical and complex designs, identification of the appropriate level for tests and full reporting of outcomes
☐	☒	Estimates of effect sizes (e.g. Cohen's *d*, Pearson's *r*), indicating how they were calculated

Our web collection on statistics for biologists contains articles on many of the points above.

Software and code

Policy information about availability of computer code

Data collection	RNA-seq by Illumina HiSeq 2500 in Rapid Run mode using 100 nt single read sequencing, a custom-designed expression microarray designed by Roche NimbleGen (Madison, WI). Proteomic data obtained by LC/MS/MS using a Waters NanoAcquity HPLC system interfaced with a Q Exactive™ Hybrid Quadrupole-Orbitrap Mass Spectrometer. CUT&Tag-seq by Illumina NEXSeq 550 using paired-end 150nt sequencing.
Data analysis	Clustal Omega for sequence alignment, I-TASSER for protein structure analysis, Scaffold for proteomic analysis, SAM for microarray analysis, SAINT for identification of associated proteins from IPs, CUfflinks, Cuffmerge, DEXseq, DEseq, and a custom-designed scripts for RNA splicing analysis, BWA and SEACR for CUT&Tag-seq analysis

For manuscripts utilizing custom algorithms or software that are central to the research but not yet described in published literature, software must be made available to editors and reviewers. We strongly encourage code deposition in a community repository (e.g. GitHub). See the Nature Portfolio guidelines for submitting code & software for further information.

Data

Policy information about availability of data

All manuscripts must include a data availability statement. This statement should provide the following information, where applicable:

- Accession codes, unique identifiers, or web links for publicly available datasets
- A description of any restrictions on data availability
- For clinical datasets or third party data, please ensure that the statement adheres to our policy

The MS raw data and peptide information were deposited to the ProteomeXchange Consortium via the PRIDE partner repository with the dataset identifier PXD032834 and 10.6019/PXD032834; Microarray data were submitted to NCBI GEO under the accession number GSE199419; RNA-Seq data were submitted to NCBI GEO under the accession number GSE199366; Custom-designed scripts for RNA splicing analysis were deposited in Zenodo under the accession number 7979503; CUT&Tag-seq data were submitted to NCBI GEO nder the accession numberGSE214535; The newly generated plasmids (pHD22Y/PfPRMT5-PTP and pHD22Y/DPfPRMT5) in this paper were deposited in Addgene with ID number 203160 and 203161, respectively. The source data behind the graphs in the paper are included in the Supplementary tables. The uncropped and unedited blot/gel images corresponding to the blot/gel images in the Figures were included as Supplementary Figures in the Supplementary information pdf file.

Research involving human participants, their data, or biological material

Policy information about studies with human participants or human data. See also policy information about sex, gender (identity/presentation), and sexual orientation and race, ethnicity and racism.

Reporting on sex and gender	N/A
Reporting on race, ethnicity, or other socially relevant groupings	N/A
Population characteristics	N/A
Recruitment	N/A
Ethics oversight	N/A

Note that full information on the approval of the study protocol must also be provided in the manuscript.

Field-specific reporting

Please select the one below that is the best fit for your research. If you are not sure, read the appropriate sections before making your selection.

☒ Life sciences ☐ Behavioural & social sciences ☐ Ecological, evolutionary & environmental sciences

For a reference copy of the document with all sections, see nature.com/documents/nr-reporting-summary-flat.pdf

Life sciences study design

All studies must disclose on these points even when the disclosure is negative.

Sample size	For most experiments, three independent biological replicates were performed. The results are presented as mean ± standard deviation (SD). Results are regarded as significant if $P < 0.05$ as established by ANOVA. To analyze the schizont numbers containing different numbers of merozoites, a $\chi 2$ goodness of fit test was first used to evaluate if the number of schizonts containing a certain number of merozoites was independent of the parasite lines. Then the proportions of schizonts with a certain number of merozoites were compared among these cell lines based on ANOVA for each merozoite number.
Data exclusions	No data was excluded
Replication	At least three independent biological replicates were performed.
Randomization	The samples were randomly chosen.
Blinding	The data were collected and analyzed blindly by the investigators.

Reporting for specific materials, systems and methods

We require information from authors about some types of materials, experimental systems and methods used in many studies. Here, indicate whether each material, system or method listed is relevant to your study. If you are not sure if a list item applies to your research, read the appropriate section before selecting a response.

Materials & experimental systems

n/a	Involved in the study
☐	☒ Antibodies
☐	☒ Eukaryotic cell lines
☒	☐ Palaeontology and archaeology
☒	☐ Animals and other organisms
☒	☐ Clinical data
☒	☐ Dual use research of concern
☒	☐ Plants

Methods

n/a	Involved in the study
☐	☒ ChIP-seq
☒	☐ Flow cytometry
☒	☐ MRI-based neuroimaging

Antibodies

Antibodies used: anti-H3R2me2s, H3R8me2s, H4R3me2s, H3, H4, H3R2me2a, HSP70, PfHLP, Protein-C, MMA, aDMA, sDMA.

Validation: anti-H3R2me2s, H3R8me2s, H4R3me2s,3R2me2a were validated by using histone without modifications. Anti-H3R2me2s was further validated by dot-blots with peptides with or without H3R2me2s modification. Anti-H3, H4, HSP70, Protein-C, MMA, aDMA, sDMA were validated from published data. Anti-PfHLP were validated by our previous publications.

Eukaryotic cell lines

Policy information about cell lines and Sex and Gender in Research

Cell line source(s): Plasmodium falcipraum 3D7

Authentication: Obtained from MR4 (ATCC)

Mycoplasma contamination: All cell lines were routinely tested and not mycoplasma contamination was found.

Commonly misidentified lines (See ICLAC register): N/A

Plants

Seed stocks: N/A

Novel plant genotypes: N/A

Authentication: N/A

ChIP-seq

Data deposition

☒ Confirm that both raw and final processed data have been deposited in a public database such as GEO.

☒ Confirm that you have deposited or provided access to graph files (e.g. BED files) for the called peaks.

Data access links *May remain private before publication.*: CUT&Tag-seq data were submitted to NCBI GEO under the accession number GSE214535.

Files in database submission: raw data of CUT&Tag-seq (BAM), Peak calling file, list of overlapped peaks.

Genome browser session (e.g. UCSC): N/A

Methodology

Replicates: Three replicates

Sequencing depth: 150 bp paired-end sequencing with 5 million reads.

Antibodies: H3R2me2s and IgG

Peak calling parameters: The peak calling was finished by SEACR with parameters 'norm' and 'stringent'

Data quality	more then 90% sequencing could be mapped to the P. falciparum genome.
Software	Mapping by using BWA after FastQC quality control, The peak calling was done by SEACR
